# Self-Applied Daylight Photodynamic Therapy: A Paradigm Shift?

**DOI:** 10.3390/ijms26020628

**Published:** 2025-01-13

**Authors:** Emilio Garcia-Mouronte, Jorge Naharro-Rodriguez, Luis Alonso-Mtz de Salinas, Luis Alfonso Pérez-González, Montserrat Fernández-Guarino

**Affiliations:** Dermatology Department, Hospital Universitario Ramon y Cajal, Carretera M-607 km 9.1, 28034 Madrid, Spain; jorgenrmed@gmail.com (J.N.-R.); lasalansa@hotmail.com (L.A.-M.d.S.); pg.l.alfonso@gmail.com (L.A.P.-G.)

**Keywords:** photodynamic therapy, ambulatory care, actinic keratosis, skin cancer, photosensitizing agents

## Abstract

Photodynamic therapy (PDT) involves the topical application of a photosensitizer and its activation by visible light, leading to the generation of protoporphyrin IX (PpIX) and reactive oxygen species. Daylight photodynamic therapy (dPDT), a variant utilizing natural sunlight as the energy source, enhances procedural flexibility by eliminating the need for specialized equipment. dPDT has been effectively used in dermatology to treat various cutaneous disorders, including neoplastic and infectious diseases. Traditionally, skin preparation and photosensitizer application are performed by trained practitioners, limiting the accessibility of dPDT for broader populations. However, recent studies suggest that these preparatory steps can be managed by patients or caregivers, enabling fully self-applied, home-based dPDT protocols. This review systematically examines the current evidence on self-applied dPDT (SA-dPDT), emphasizing molecular mechanisms and its efficacy in managing premalignant and other cutaneous conditions.

## 1. Introduction

PDT is a minimally invasive therapeutic technique in which a photosensitising compound selectively accumulates, in vivo, within a target tissue. Subsequent activation of the photosensitiser with a light source of an appropriate wavelength (in dermatology, red light from the visible spectrum at approximately 630 nm is preferred) triggers the production of reactive oxygen species (ROS). These ROS exert cytotoxic effects on tumour cells [1,2].

Its primary application in dermatology is in the treatment of non-melanoma skin cancer (NMSC), field cancerisation (FC), non-hypertrophic actinic keratoses (AKs), Bowen’s disease (BD), and superficial basal cell carcinoma (sBCC). Since its introduction in the 20th century, PDT has also been successfully employed for a range of other dermatological conditions, including bacterial and fungal infections, cutaneous leishmaniasis (CL), warts, acne, hidradenitis suppurativa, psoriasis, morphea, alopecia areata, lichen sclerosus, and Kaposi’s sarcoma, among others [1,2].

PDT boasts an excellent safety profile, being minimally invasive and effective in treating both clinically evident and subclinical FC lesions. It achieves superior aesthetic outcomes compared to surgery and can be combined with other treatment modalities. Despite its versatility, conventional PDT (cPDT) has notable limitations, including significant pain during the procedure, which can discourage patients from completing treatment or undergoing additional sessions, as well as requiring substantial time, technical resources, and trained personnel [3].

To address these challenges, alternative PDT modalities have gained attention in recent years. Among these, dPDT, combined PDT (which integrates conventional and daylight PDT), and intralesional PDT (in which the photosensitiser is administered via hypodermic needles or specific devices directly into the lesion, enhancing penetration and enabling treatment of thicker lesions) are particularly noteworthy.

Daylight photodynamic therapy (dPDT) is currently one of the most widely used PDT modalities alongside cPDT. Its primary distinction lies in the use of natural sunlight as the light source, which has significantly lower intensity compared to the artificial lamps used in cPDT. This slower activation of PpIX results in minimal pain. dPDT utilises the entire visible spectrum of sunlight (400–800 nm), with an estimated light dose of 4–8 J/cm² considered effective for successful treatment. To mitigate the harmful effects of UV radiation on photosensitised tissue, particularly UVB, the use of a topical photoprotector with an SPF of 30–50 is recommended, or alternatively, conducting dPDT indoors where windows filter out UVB radiation [1,2].

Unlike cPDT, dPDT involves non-occlusive topical application of the photosensitiser and a shorter incubation period, reduced from 2–3 h to a maximum of 30 min, aiming for slower and less intense photosensitisation. Following incubation, patients are exposed to sunlight for approximately two hours, although protocols may vary from 90 min to 4 h. Although the sunlight exposure typically lasts around two hours, the overall treatment duration for dPDT is comparable to cPDT due to its shorter photosensitiser incubation times. This leads to reduced hospital time and associated costs [1].

However, the ageing population in Western countries introduces additional challenges. Candidates for PDT often have cognitive or mobility impairments, multiple comorbidities, and fragmented medical care involving several specialists. For these patients and their caregivers, even short clinical visits, such as those required for dPDT preparation, can be burdensome. In this context, SA-dPDT presents an innovative solution, enabling patients to independently perform dPDT in a home-based setting. This approach has the potential to enhance accessibility and convenience while maintaining therapeutic efficacy [1].

This review represents the first published analysis of SA-dPDT. The primary objective is to evaluate the current scientific evidence on the effectiveness, tolerability, and safety of fully home-based dPDT protocols, particularly in managing premalignant and inflammatory skin conditions, with a focus on AK.

## 2. Mechanisms of Action

PDT relies on a photochemical reaction involving a photosensitising agent, light exposure, and oxygen to induce oxidative stress, resulting in the selective destruction of abnormal tissue [4,5].

### 2.1. Photosensitisers

The photosensitising properties of certain compounds were first identified in the early 20th century. In 1908, Hausmann demonstrated the phototoxic effects of haematoporphyrin (HP) in animal models. In 1913, Friedrich Meyer-Betz self-administered HP intravenously, experiencing prolonged phototoxic reactions lasting two months [6,7]. In 1995, Schwartz isolated haematoporphyrin derivatives (HPDs), which demonstrated selective tumour accumulation and cytotoxic effects in breast and urinary cancer [7,8]. Shortly afterward, porfimer sodium (Photofrin^®^) was the first photosensitizer approved by the FDA, in this case for malignant bladder tumours [6].

Porphyrin, a derivative of HPDs, became the first photosensitiser approved by the FDA specifically for cutaneous conditions. However, it exhibited significant limitations, including persistent phototoxicity following intravenous administration [6].

Currently, second-generation topical photosensitisers, such as 5-δ-aminolevulinic acid (ALA) in nanoemulsion form (BF-200 ALA; Ameluz^®^, Biofrontera, Leverkusen, Germany) and methyl-aminolevulinate (MAL; Metvix^®^, Galderma, Montdésir, France), are widely used [1,7,8]. These new photosensitisers are chemically pure, produce minimal toxicity in the absence of light, are non-mutagenic, selectively accumulate in tumour cells, have a high molar extinction coefficient in the red light region of the visible spectrum (600–800 nm), exhibit short half-lives with rapid tissue clearance, and maintain high photodynamic efficiency with sufficient ROS production [1,7,8].

ALA is an intermediate metabolite in the heme biosynthesis pathway. Under normal conditions, heme synthesis begins in the cytoplasm of the cell with the reaction of glycine and succinyl-CoA, catalysed by the enzyme ALA synthase. Subsequently, ALA is metabolised by ALA dehydratase to produce porphobilinogen. Further enzymatic actions (porphobilinogen deaminase and uroporphyrinogen synthase) combine four porphobilinogen molecules to form uroporphyrinogen III, which is converted into coproporphyrinogen III by uroporphyrinogen decarboxylase. This molecule enters the mitochondria, where it is transformed into protoporphyrinogen, the direct precursor of protoporphyrin IX (PpIX). Finally, ferrochelatase acts on PpIX to form heme [9,10].

Exogenous administration of ALA or MAL leads to overproduction and accumulation of mitochondrial PpIX, which is responsible for tissue photosensitisation [9,10]. In cPDT, a 2–3 h incubation period allows sufficient PpIX accumulation. Tumour cells preferentially accumulate more PpIX due to the increased activity of enzymes such as ALA hydratase and uroporphyrinogen decarboxylase, along with decreased ferrochelatase activity [9,10].

### 2.2. Light Source

The efficacy of PDT largely depends on the light source (wavelength, fluence, exposure time, and illumination area) and the absorptive properties of the target tissue (absorption coefficient). Effective PDT requires matching the light source wavelength to PpIX absorption peaks. While PpIX’s highest absorption occurs at 405 nm (Soret band), this wavelength has limited tissue penetration due to the rapid loss of fluence as light penetrates tissue, with competition from molecules such as melanin and haemoglobin. Red light (RL) at 635 nm, with greater tissue penetration (4–6 mm compared to 1–2 mm with blue light (BL)), is therefore preferred in most dermatological indications due to its lower energy and reduced affinity for melanin and haemoglobin [9,10].

In cPDT, specific light sources, such as broad-spectrum lamps, diode lamps, or lasers, activate the photosensitiser, enabling rapid healing and cosmetic benefits for conditions like AK, sBCC, and BD [5]. Alternatively, dPDT is primarily indicated for thin AK and FC, offering fewer adverse effects, reduced pain, and lower rates of treatment discontinuation [8].

### 2.3. Biological Effects

PDT biological effects can be summarised in three key processes:

- Direct tumour cell destruction: ROS generated during PDT cause oxidative stress, leading to cellular damage, apoptosis, or necrosis, eliminating cancer cells [11,12,13,14,15].
- Immune system activation: PDT induces immunogenic cell death, stimulating the release of tumour antigens and enhancing immune response, aiding in targeting remaining tumour cells and distant metastases [16,17,18].
- Vascular disruption: PDT damages blood vessels supplying the tumour, reducing oxygen and nutrient delivery, which results in tumour cell death due to ischemia [19].

#### 2.3.1. Direct Phototoxic Effects

Direct tumour cell destruction is the primary contributor to tumour damage. Upon light activation, PpIX transitions from a ground state (S0) to an excited singlet state (S1) with a short half-life [11]. The molecule may then return to its ground state, through fluorescence emission or internal conversion (without fluorescence release), or proceed to a longer-lived less energetic triplet state [11]. In the triplet state, it transfers energy to intracellular oxygen, generating singlet oxygen (¹O₂), a highly ROS responsible for inducing cell death [11].

**Figure 1:**
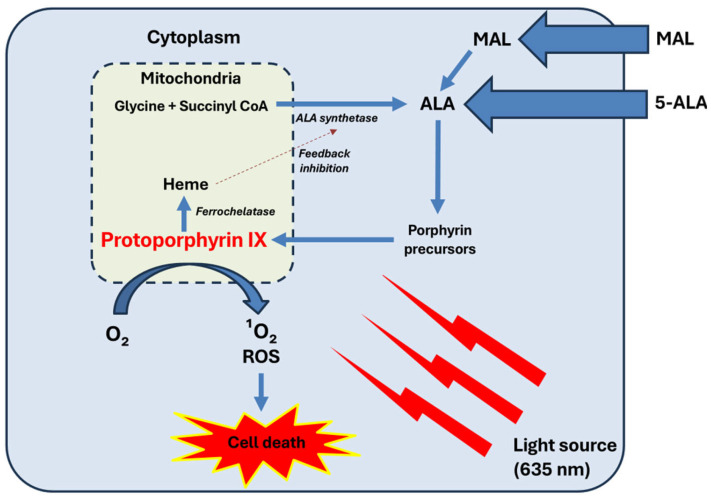
Mechanism of action of PDT. Exogenous ALA or MAL are taken up by cells. MAL needs to be hydrolysed to ALA by intracellular esterases. ALA enters the heme biosynthesis pathway. Within the mitochondria, glycine and succinyl-CoA are converted into ALA by the enzyme ALA synthetase. This step is tightly regulated by negative feedback from heme, preventing excessive production of intermediates. ALA progresses through the porphyrin biosynthetic pathway, eventually forming PpIX. In normal conditions, PpIX is converted into heme by ferrochelatase. However, in tumour cells, reduced ferrochelatase activity leads to the preferential accumulation of PpIX. Upon exposure to a light source (usually at 635 nm), PpIX becomes activated, leading to the generation of singlet oxygen (¹O₂) and other ROS, such as superoxide anion radicals (O2•−), hydrogen peroxide (H_2_O_2_), and hydroxyl radicals (•OH). These ROS induce oxidative damage to cellular structures, resulting in selective tumour cell death.

In particular, the singlet oxygen causes defects in the tumour cell membrane, altering its integrity and affecting transport functions between the intracellular and extracellular environments. Additional damage occurs in the membranes of the nucleus, mitochondria, lysosomes, and endoplasmic reticulum [12,13,14]. Fluorescence microscopy studies suggest that mitochondrial phototoxicity is the primary cause of cell death induced by PDT [12,13]. Regardless of the exact cytotoxic target, the outcome is a loss of cellular integrity, release of inflammatory mediators, and activation of the complement cascade, leading to the tumour cell death (Figure 1) [10,14,15].

#### 2.3.2. Immunological Effects

PDT induces a robust immune response by releasing damage-associated molecular patterns (DAMPs) [16,17]. These DAMPs act as “danger signals” that activate key components of the immune system, including neutrophils, macrophages, and dendritic cells. These cells play pivotal roles in the early stages of the immune response [18].

Following PDT, immune cell infiltration into the tumour and surrounding tissue is predominantly driven by neutrophils. DAMPs facilitate this by aiding the presentation of tumour antigens on dendritic cell surfaces via major histocompatibility complexes, a critical step for activating the adaptive immune response and triggering T-cell activity [20,21].

Cytotoxic CD8+ T lymphocytes are key players in targeting and eliminating tumour cells that escape the immediate phototoxic effects of PDT. Concurrently, helper CD4+ T-cells are activated and release pro-inflammatory cytokines such as interleukin-1 beta (IL-1β), tumour necrosis factor-alpha (TNF-α), and interleukin-6 (IL-6). These cytokines enhance the recruitment and activation of additional immune cells, amplifying the antitumour response [22,23,24].

B-cells also contribute to the immune response by producing antibodies against tumour-associated antigens, facilitating immune recognition and aiding in tumour clearance [22,23,24]. The synergy between innate and adaptive immunity, stimulated by PDT, enhances both local and systemic antitumour effects, offering long-term therapeutic benefits [22,23,24].

#### 2.3.3. Vascular Effects

PDT exerts significant effects on the microvasculature by targeting endothelial cells and the vascular basement membrane, initiating thrombogenic processes. This cascade leads to platelet aggregation, the release of vasoactive molecules, leukocyte adhesion, increased vascular permeability, and vessel constriction. These events culminate in tumour destruction via vascular collapse and haemorrhage, effectively cutting off the oxygen and nutrient supply to the tumour [19].

## 3. Treatment Protocol

While specific details may differ between studies, the protocol for SA-dPDT shares several fundamental components as summarized below. These commonalities provide a consistent approach to effectively applying SA-dPDT and facilitating treatment success.

### 3.1. Skin Preparation

Preparation of the skin is a critical step to ensure effectiveness. In the case of AK, patients are instructed to prepare the skin by applying a keratolytic agent, such as 30% urea cream or 10% salicylic petrolatum, for 7 days before the treatment. This step helps remove scales and crusts, improving the penetration of the photosensitizer [1,25].

On the treatment day, the target area might be roughened using an abrasive pad to further enhance drug absorption. In some cases, skin preparation can be done by a healthcare provider at an initial visit, using curettage. Sunscreen with sun protector factor (SPF) 50+ should be applied 15 min before the application of the photosensitizer to protect surrounding areas from unintended exposure to daylight [26]. The photosensitizer, either MAL or BF-200 ALA gel, is then applied in a thin layer to the treatment area [27,28].

### 3.2. Patient Instructions

Clear and thorough instructions are crucial for the success of SA-dPDT. Patients should receive both oral and written instructions, which may include visual aids like infographics to simplify the process. The instructions should detail the previously detailed preparation steps [27,28].

Patients or their caregivers must perform by themselves the following steps:

1. Administration of a sunscreen of SPF 50+ with organic filters in all sun-exposed areas.
2. Roughening of the targeted area with an abrasive pad, to remove remaining crusts and scales.
3. Application of a thin layer of the prodrug, using nitrile finger gloves and avoiding contact with mucous membranes.
4. Within 30 min of the photosensitizer application, self-direct exposure to natural daylight for 2 h.

The treatment can be adjusted based on weather conditions, allowing for time in the shade if discomfort occurs on sunny days. After the procedure, the cream will be washed off, and further sun exposure will be avoided for the next 48 h. Protective measures, such as photoprotective clothing and hats as well as SPF 50+ sunscreen on treated areas are encouraged [1,27]

**Figure 2:**
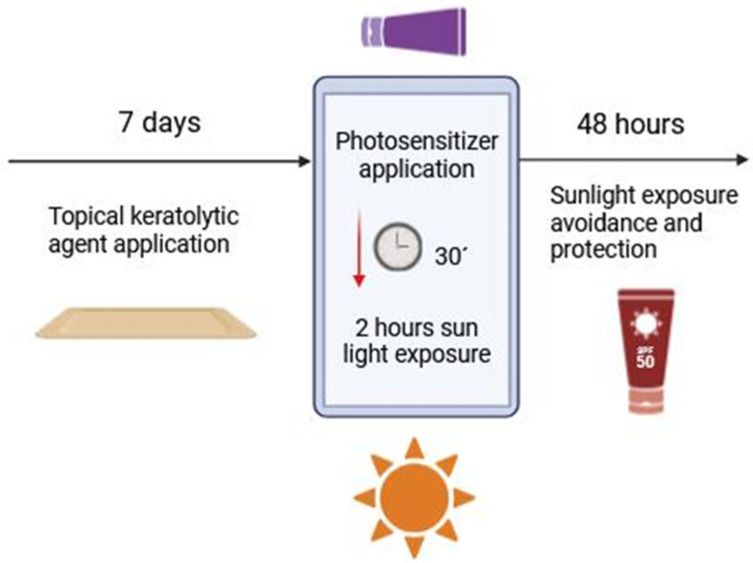
Schematic representation of a standard SA-dPDT protocol.

Patients should also be provided with a schedule to record treatment details, including the time of each step, and should be contacted by the healthcare team within 24 h to assess any local skin reactions and ensure adherence to post-treatment care [26]. A schematic version of the SA-dPDT protocol is shown in Figure 2.

## 4. Self-Applied Daylight Photodynamic Therapy for Actinic Keratoses

### 4.1. Actinic Keratoses: Clinical Presentation and Molecular Aspects

Actinic keratoses (AKs) are the most common form of keratinocytic intraepithelial neoplasia (carcinoma in situ), typically developing on chronically sun-damaged areas such as the face, scalp, neck, and dorsal aspects of the extremities [1,29,30,31,32]. The pathogenesis of AKs is complex, involving cumulative ultraviolet B-induced disruptions in cellular proliferation, differentiation, and local immunosurveillance, leading to impaired tissue remodelling, oxidative stress, and impaired apoptosis [33,34]. Several mutations have been found in key genes for malignant transformation, such as p53, p16INK4a, bcl-2, cyclin D1, H-Ras, and PTEN, among others [32,35,36,37].

The incidence of AK is higher in male and elderly individuals and immunosuppressed hosts [38]. Without treatment, AK lesions may follow one of three possible courses [29]:

- Spontaneous involution: Regression rates vary significantly across studies, with annual rates typically reported between 15% and 63% [29,39,40]. However, up to 50% of regressed lesions may recur within the first year [40].
- Stable persistence.
- Progression into cutaneous squamous cell carcinoma (SCC). Although the malignant transformation rate per lesion-year is low (0–0.075%), risk factors such as male sex, fair phototype, history of NMSC, extensive FC, immunosuppression, therapy resistance, and advanced age elevate the risk. In high-risk individuals, transformation rates can reach up to 16% per year [29,32,41,42,43,44,45].

AK lesions vary in clinical presentation, with characteristics including hyperkeratosis (*stratum corneum* thickening), parakeratosis (presence of nucleated keratinocytes in an abnormal *stratum corneum*), epidermal thickness (ranging from atrophy to acanthosis), erythema, pigmentation, and inflammation [29,31,32,46]. Lesion size typically ranges from a few millimetres to several centimetres, and lesions often present as multiple papules rather than solitary lesions [47,48].

Macroscopic lesions are surrounded by subtle peripheral changes exhibiting genetic alterations similar to those found in AK [29,49,50,51,52,53]. This phenomenon, termed “field cancerization” (FC), was first described by Slaughter et al. [54] in 1953 in the context of oral SCC. Clinically, FC is characterised by at least two of the following features: telangiectasia, atrophy, mottled pigmentation, laxity, and a rough, emery-like texture [32,55,56]. However, there is no universal consensus on the definition of FC, and the existing ones do not comprehensively address molecular, histological, and immunological aspects [32].

### 4.2. Actinic Keratoses: Classification Systems

Various classification systems have been developed to standardise clinical trials and predict the aggressive potential of AK [29]:

- Olsen classification. This system categorises AK lesions into three subtypes based on the their thickness [30,57]:
  ◦ Grade I: Erythematous and keratotic patches that are barely visible and have a characteristic sandpaper texture.
  ◦ Grade II: Erythematous, scaly patches of moderate thickness that are easily palpable and visible.
  ◦ Grade III: hyperkeratotic patches, which may present as cutaneous horns. Crust removal and dermoscopic evaluation may be necessary to differentiate from other conditions, such as BD and SCC [31].
- Röwert-Huber classification [58]. AK lesions are subdivided into three stages based on the upwards growth pattern and extent of atypical keratinocytes within the epidermis, in analogy to human papillomavirus-induced intraepithelial neoplasias:
  ◦ AK I (mild atypia): Dysplastic cells with enlarged, pleomorphic, and hyperchromatic nuclei are confined to the lower third of the epidermis. These cells lack a basaloid phenotype and have a high nuclear/cytoplasmic correlation.
  ◦ AK II (moderate atypia): Atypical keratinocytes extend to the two lower thirds of the epidermis.
  ◦ AK III (severe atypia): Full thickness atypia with complete replacement of normal keratinocytes.
- PRO classification [59]. Established by Schmitz and colleagues, this system focuses on basal proliferation and the downward growth of atypical keratinocytes:
  ◦ PRO 0: No appreciable basal proliferation.
  ◦ PRO I (crowding): Hyperplasia of atypical keratinocytes, densely packed within the stratum basale, leading to increased basophilia and polarity loss.
  ◦ PRO II (budding): Small, hemispherical buds of dysplastic keratinocytes slightly protrude into the papillary dermis, forming round nests.
  ◦ PRO III (papillary sprouting): Atypical keratinocytes grow in elongated, spiky projections, exceeding the thickness of the overlying epidermis.
- Actinic Keratosis Field Assessment Scale (AK-FAS) [60]. Conceived by Dréno and colleagues [60], this evaluation system scales head-and-neck FC severity into five grades based on the extent of the AK area, the degree of hyperkeratosis, and sun damage severity.
- Actinic Keratosis Area and Severity Index (AKASI) [61]. Created by Dirschka et al., this system evaluates the entire FC rather than individual lesions, assessing various parameters such as sun-damaged skin extent, distribution, erythema, and lesion thickness.
- Werner classification [62]. The most practical, office-oriented system, classifying patients based on disease burden as follows:
  ◦ Single AK: ≤4 AK lesions in a single defined field.
  ◦ Multiple AK: ≥5 AK lesions in a single defined field.
  ◦ FC: ≥6 AK lesions in a single defined field, with adjacent sun-damaged skin.
  ◦ AK with immunosuppression.

However, these systems lack prognostic validation and practicality in clinical settings, as lesions with different grades can coexist within the same FC, and classification scores are generally not incorporated into routine clinical workflows or linked to actionable clinical consequences [29,31,32].

Recent studies suggest that the sequential progression model assumed by the Röwert-Huber classification may not fully capture AK behaviour; for instance, AK III lesions are not always associated with the highest risk of SCC transformation [63]. Instead, AK I lesions but with advanced basal proliferation (PRO III) are found often near SCC [63].

Interestingly, no correlation has been established between clinical and histological severity in AKs [58]. Schmitz and colleagues [30] reported a discrepancy between the Olsen and Röwert-Hubert classification systems of 46.2% in 892 head-and-neck AK lesions, with a low Spearman’s rank correlation coefficient (*r* = 0.0499, *p* = 0.137).

Histological classification alone is impractical for general use, as biopsies carry some risk of complications and should be reserved for lesions with equivocal diagnoses, therapy-resistant lesions, or those suspected to be SCC due to rapid growth, bleeding, pain, or ulceration [32,49,61,64]. Additionally, biopsy results from a single lesion do not provide a comprehensive assessment of the histopathologic severity across the full FC area [30].

### 4.3. Treatment Options for Actinic Keratoses

Since it is impossible to accurately predict the progression of each individual AK lesion within a single patient, current guidelines take a precautionary approach and recommend careful monitoring and treating, if feasible, of all AK lesions and the entirety of affected FC areas [31,46,61,62,65,66]. The primary goal of AK management is preventing malignant transformation into SCC [49,67].

AK imposes a significant financial burden on healthcare systems globally, largely due to its high prevalence (11–25% in the United States) [68], chronic and relapsing nature, and risk of progression to invasive carcinoma [29,69].

As there is no universally accepted gold-standard treatment, multiple factors should be considered when tailoring the optimal therapeutic regimen for each patient. These factors include the number of lesions, clinical presentation, anatomical location, comorbidities, concurrent medications, ease of use, treatment duration, anticipated tolerance and compliance, past treatment experience, and the patient’s needs and preferences [31,32,46,49,62]. When multiple lesions (≥5) or FC changes are present, topical therapies should be prescribed for subclinical damage across the entire affected area, reducing the risk of recurrence and malignant transformation [31,32,46,48,49,62].

Despite the wide array of availabel treatments, no approach has yet demonstrated sustained long-term clearance [29]. Consequently, most patients require sequential treatments over time to manage lesion recurrence [29].

With the expected increase in the aging population and cumulative lifetime sun exposure, the prevalence of AK is projected to rise substantially, particularly among the elderly population [29]. Identifying therapeutic regimens that are well tolerated, easy to apply, and yield rapid results is crucial for this population, as most licensed treatments are associated with local cutaneous reactions, including erythema, crusting, blistering, erosions, and even bleeding [29,31,32,46,49].

The licensed FC-targeted treatments in Europe include the following: 5-fluorouracil cream, imiquimod cream, tirbanibulin ointment, diclofenac sodium gel, cPDT, and dPDT [70,71,72,73].

### 4.4. Mechanisms of Action of Photodynamic Therapy in the Management of Actinic Keratoses and Cancerization Fields

PDT not only targets surface lesions but also addresses deeper molecular changes induced by chronic ultraviolet UV exposure.

Some studies have explored the immunohistochemical changes in DNA damage and proliferation markers such as p53, Ki-67, and cyclin D1 after PDT. Abdalla et al. [74] reported that 92.86% of treated AK samples and 75% of perilesional skin still showed positive p53 staining, indicating residual genetic damage that may not be addressed by a single session. Similarly, Bagazgoitia et al. [75] noted a significant reduction in Ki-67 levels, a marker of cellular proliferation, but observed that cyclin D1 expression remained unchanged. This suggests that while PDT can reduce proliferative activity, the underlying cell cycle dysregulation may persist, requiring additional treatments to achieve complete molecular resolution.

The impact of PDT on the extracellular matrix (ECM) is a critical component of its therapeutic effect, as noted in studies by Szeimies et al. [76] and Almeida Issa et al. [77]. Procollagen-I, a precursor of collagen type I, is essential for dermal repair and structural integrity. Following PDT, an increase in procollagen-I expression was observed, indicating enhanced collagen synthesis. This is particularly beneficial in reversing the effects of UV-induced photoaging, which typically results in decreased collagen production due to heightened matrix metalloproteinase (MMP) activity [76,77].

The role of MMP-1, a collagen-degrading enzyme, was also highlighted. Elevated MMP-1 levels post-PDT suggest an initial phase of ECM breakdown, likely aimed at removing damaged collagen fibres and facilitating the subsequent synthesis of new collagen. However, the increase in MMP-1 was not statistically significant across all patients, indicating variability in the remodelling response [76]. Furthermore, the study by Almeida Issa et al. reported consistent increases in MMP-9 and procollagen-I after three months of MAL-PDT, reinforcing the idea of active tissue remodelling and repair [77].

Tenascin-C (Tn-C), an ECM glycoprotein associated with tissue repair, inflammation, and even tumour progression, was found to be significantly upregulated after multiple PDT sessions. Szeimies et al. [76] noted that Tn-C expression increased in the papillary dermis, which is indicative of the skin’s response to the controlled injury induced by PDT. The elevated levels of Tn-C likely reflect ongoing ECM reorganization and support the formation of a more resilient dermal matrix [76]. This remodelling effect is supported by the observed upregulation of genes involved in ECM synthesis, suggesting improved skin structure and the potential for photo-rejuvenation [78].

Joly et al. [79] conducted an in-depth analysis of gene expression changes before and after MAL-PDT, using biopsies from AK lesions, peri-lesional skin, and unaffected areas. The results showed that PDT corrected the dysregulated expression of genes implicated in cancer progression, particularly those involved in epidermal differentiation, cell cycle control, and DNA repair. The normalization of these genes implies a reversal of abnormal keratinocyte proliferation, reducing the potential for malignant transformation. Genes associated with tissue repair and ECM synthesis, including COL1A1 and COL3A1, were significantly upregulated, supporting the observed clinical improvements in skin texture and elasticity.

Finally, the presence of TERT (telomerase reverse transcriptase) promoter mutations has been identified as a key factor in the early stages of tumorigenesis in AK. Srinivas et al. [80] assessed the prevalence of these mutations in AK lesions before and after dPDT treatment, alongside the expression of p53. Before treatment, TERT promoter mutations were found in 20.8% of lesions, reflecting a significant mutational burden associated with increased telomerase activity. However, post-treatment analysis revealed a substantial decrease, with only 1.9% of lesions retaining TERT mutations. Additionally, p53 expression and histological signs of dysplasia were significantly reduced after dPDT [80].

### 4.5. Photodynamic Therapy for Actinic Keratoses: Treatment Protocols

PDT protocols use commercially availabel light-sensitizing compounds applied either entirely or partially to the affected area, followed by exposure to a source of non-coherent visible light energy, which can be artificial (e.g., RL or BL) or natural (sunlight) [31,32,46,49,81]. The most commonly used photosensitizers are ALA in forms such as 10% or 20% nanoemulsion gel or self-adhesive patches, and MAL in a 16% cream formulation [1,31,32,46,49,82].

The most recent American and European guidelines for using of PDT in AK and FC management are summarized in Table 1 [31,32,46,49].

dPDT offers a nearly painless alternative to cPDT with comparable efficacy, fewer adverse events, and a simpler application process, as it eliminates the need for the incubation (up to 3 h) and occlusion of treated sites [1,4,49,71,81,82,83,84,85,86,87,88,89,90,91]. Using natural light as an energy source increases flexibility, removes the requirement for specialised illumination equipment, and allows for quicker, single appointments, as well as treating larger and even non-contiguous FC areas as compared to cPDT [4,81,92,93,94,95].

dPDT is indicated for treating multiple mild-to-moderate non-hypertrophic AK lesions (Olsen grades I and II) in areas that can be readily exposed to illumination [1,4,81,82,92,93,94,96]. The main candidates are patients whose predicted tolerance to acute side effects of FC-targeted therapies is poor [1,81,82,84,92,93,94,96]. dPDT could also be an optimal choice for patients with a history of residual dyschromia following AK-targeted ablative therapies (i.e., cryotherapy) [93].

Interestingly, the European Dermatology Forum guidelines on topical PDT highlighted in 2019 the potential of home-based MAL-PDT in the management of AK, resulting in good efficacy and tolerability, with high levels of patient satisfaction [82].

### 4.6. Self-Applied Daylight Photodynamic Therapy for Actinic Keratoses: Current Evidence

The scientific evidence on home-based or SA-dPDT for managing AKs and FC is summarized in Table 2 [26,27,28,67,97]. To date, there are four observational studies [26,28,67,97] and one randomised clinical trial (RCT) [27] in the medical literature. Most studies were pilot trials with small sample sizes ranging from 9 [67] to 50 [28] participants. Overall, 109 patients have been treated with SA-dPDT, most of whom were male (86.10%) and elderly (mean age: 72.14 ± 4.25 years) [26,27,28,67,97]. The majority of participants (68.2–100%) had previously received at least one treatment for AKs, with cryotherapy being the most common (15–66.67%) [26,28,67]. Collectively, more than 880 head-and-neck AK lesions were treated using home-based dPDT protocols [26,27,28,67]. While all observational studies used MAL as the photosensitizer [26,28,67,97], BF-200-ALA was utilised in the single non-inferiority RCT [27].

Surface skin preparation and scale removal were fully performed by patients or their caregivers in two studies [26,27], using topical keratolytic products (urea 30% cream or 10% salicylic petrolatum) applied once a day for a week before the first dPDT session. In Levi et al. [67], an initial gentle curettage was performed by a practitioner, after which subsequent sessions allowed patients to opt for a fully home-based protocol. Karrer et al. [28] limited curettage to a third of participants, focusing only on single hyperkeratotic lesions.

For the remaining protocol steps, all groups adhered strictly to manufacturer guidelines, consensus documents, and established recommendations [26,27,28,67,97]. Three out of five studies were conducted in Mediterranean countries (Spain and Israel), benefiting from longer, sunnier seasons conducive to increased daylight exposure possibilities [26,27,67]. Participants received between one and five dPDT sessions, depending on individual needs [27,28,97].

Clinical outcomes varied and were measured by lesion clearance, lesion count, and reductions in severity indices (i.e., AKASI, AKQoL) [26,27,28,67,97]. Three months after the last SA-dPDT session, AK lesion reduction changed from 62% to 82.1% [27,28]. Follow-up periods varied widely, from 3 months [27,28] to nearly 3 years [67].

Adverse skin reactions were common but mild and transient, typically requiring no medical intervention [26,27,28,67]. Pain was assessed in three out of the five studies, using either a numeric rating scale (NRS) or visual analogue scale (VAS), with a mean intensity of 1.14 ± 0.45 [26,27,28]. Patient satisfaction was consistently high across all studies [26,27,28].

Regarding the particular works, in 2018 Levi et al. [67] were the first group of investigators to assess the effectiveness and tolerability of the SA-dPDT protocol in the management of premalignant cutaneous disorders, specifically actinic cheilitis (AC) (Table 2). This is the mucosal equivalent of AK, frequently involving the vermillion border of the lower lip [67,98]. This observational study included nine patients with a histologically confirmed AC, most of whom were male (66.67%) and middle-aged (60.11 ± 14.93 years) [67]. After an initial practitioner-administered MAL-dPDT session involving gentle curettage and photosensitizer application, patients received 2.5 h of sunlight exposure. Following the first session, patients could choose between practitioner- and self-applied sessions; 81.81% opted for the latter, showing a preference for at-home care [67]. Participants received subsequent MAL-SA-dPDT sessions at 2- to 4-week intervals after clinical complete remission was achieved, with a mean number of sessions of 2.89 (2–6) [67]. No relapses were detected over a long follow-up period (31.44 ± 18.58 months) [67]. This excellent response was striking, as all AC lesions had been refractory to multiple previous treatments [67].

Despite these promising results, the authors’ conclusions should be considered carefully. As all patients received both practitioner- and SA-dPDT sessions, it was impossible to discern whether there was a difference at all in terms of clinical efficacy and safety between these two modalities [67]. Histological evaluation of response was only performed in 45% patients, raising the possibility of whether the unassessed participants had persistent dysplastic alterations that could progress into clinical relapses months or years after treatment cessation [67].

One year later, Karrer et al. [28] conducted the largest interventional study on MAL-SA-dPDT, involving 90 participants with non-hypertrophic AK on the head and scalp (Table 2).

Clinical response at the 3-month follow-up visit was considerably good, with a mean lesion clearance of 62% [28]. One of the greatest strengths of this study is that it extensively analysed patient-reported outcomes [28]. All patients (100%) confirmed they had received enough detailed instructions to correctly perform the procedure [28]. Nearly every participant closely adhered to the instructions (98–100%) and considered them very convenient (98%) [28]. Overall patient satisfaction was high in participants both immediately and 3 months after the treatment session (98% and 96%, respectively) (Table 2) [28]. In this respect, of those patients having previously received at least one treatment for their AK, 78% considered MAL-SA-dPDT to be better than the previous therapy and 85% affirmed they would use MAL-SA-dPDT [28].

Tolerability was excellent, as maximum pain felt during illumination was minimal (NRS: 1 ± 1.4) (Table 2) [28]. Half of the patients reported no pain at all [28]. Additional side effects such as erythema were mild and transient, with a self-reported mean duration of downtime of 2.8 days [28]. It should be noted that the utilised MAL-SA-dPDT protocol was not strictly fully home-based in a third of the participants, as a first removal of the scales was still performed by the investigator through in-patient curettage or skin abrasive pads 1 week prior to the application of the photosensitizer [28]. Nevertheless, the impact of this manoeuvre on the overall validity of the study is minimal, as the practitioner-performed preparation was confined to a single individual lesion in 75% of the cases [28].

This limitation was totally overcome by Garcia-Gil et al. [26], as they designed the first prospective observational study evaluating the efficacy and safety of a fully home-based MAL-SA-dPDT protocol in the management of AK (Table 2). A total of 22 participants with AK on the head and/or scalp, mostly male (90.5%) and elderly (72.05 ± 6.96 years), were treated with at least one MAL-SA-dPDT session [26]. Baseline severity was remarkable, with an mean lesion count and AKASI score of 12.5 ± 4.34 and 4.99 ± 2.43, respectively [26]. The majority of patients had received at least one previous treatment for their condition (68.2%), with diclofenac being the most common FC-targeting therapy (27.3%) [26]. Interestingly, 4.5% of participants had already been treated in the past with clinic-based dPDT [26].

Participants were provided with oral and written instructions on how to perform the SA-dPDT protocol, and most (90.9%) of them were satisfied with their clarity [26]. Skin surface preparation was performed through self-application of a urea 30% cream in the treated area, once a day for one week before the application of the photosensitizer [26]. Adverse reactions were reported in 72.7% of participants but were mild and transient, mainly consisting of erythema (31.8%) and crusts (22.7%) [26]. Reported pain during sunlight exposure was minimal (0.9 ± 2.32) (Table 2) [26].

Of note, 86.36% required an additional MAL-SA-dPDT session during the follow-up period to control the disease, mostly at the 3-month visit [26]. At this time point, patients’ satisfaction with overall outcomes was moderate (72.7%). One year after the initial procedure, a statistically significant reduction (*p* = 0.0234) in the FC severity assessed through the AKASI score was observed (−46.67%), although this did not correlate with an improvement in the quality of life of the patients (*p* > 0.05) [26].

This work obviously has several limitations related to its observational nature [26]. Although this new variant of dPDT was not compared to any other treatment and the clinical assessment was unmasked, it is remarkable that some of the patients had significant risk factors that increased their resistance to treatment and the risk of progression into SCC, such as active immunosuppression (13.6%) and a personal history of NMSC (45%) (Table 2) [26]. Incorporating these high-risk profiles in the investigation of new treatment modalities for AK is essential, as these patients are those in the highest need for effective, safe, and fast therapeutic strategies and are unfortunately usually excluded from RCTs.

O’Reilly et al. [99] presented a proof-of-concept of MAL-SA-dPDT protocols in a community setting of 17 participants with head-and-neck non-hypertrophic AK (Table 2). After selection by dermatologists, participants were provided with nurse-led training and a fully recyclabel home kit prior to the SA-dPDT session [99]. Further sessions were prescribed if deemed clinically appropriate [99]. Compared to other observational studies, patients in this cohort showed a higher treatment resistance, with a mean number of sessions of four [99]. A third of the patients had a bad response, with a lesion clearance lower than 50% (Table 2) [99]. The authors concluded that adequate individualization and patient selection was critical for therapeutical success [99]. As the sessions yielded unsatisfactory outcomes in FC with hyperkeratosis, the authors concluded MAL-SA-dPDT protocols should be restricted to milder cases [99].

After these observational studies, Saenz-Guirado and colleagues [27] published the first and only RCT assessing the efficacy of a home-based BF-200-ALA-PDT protocol in the management of head-and-neck AK (Table 2). Most participants were male (95%) and elderly, with a mean age of 79.25 ± 5.86 years [27]. Approximately two out of three AK lesions were located on balding scalps [27]. The baseline AK count was moderate in both groups (12.09 ± 4.16 vs. 13.56 ± 3.79), without statistically significant differences (*p* = 0.425) [27].

At the baseline visit, AK Olsen grade III was treated with two cycles of 10 s cryotherapy [27]. Regarding skin surface preparation, no curettage was performed, and participants in both groups applied 10% salicylic petrolatum once a day for 7 days in the targeted area prior to the treatment onset [27]. In this respect, clinic-based and home-based protocols only differed with regard to whom applied and removed the topical photosensitizer [27].

Home-based and clinic-based protocols proved to be effective in the treatment of AK and FC (Table 2) [27]. At the 3-month follow-up visit, the overall AK reduction rate was similar in the two groups (82.1% vs. 71.58%, *p* = 0.19) [27]. No statistically significant differences were found in terms of remaining AK (2.36 ± 1.91 vs. 3.56 ± 2.45) or new lesions (0.45 ± 1.51 vs. 1.11 ± 1.27) [27]. Thickness of the lesion did not seem to impact the response, as the subgroup analysis showed clinical outcomes were similar between AK for Olsen grades I and II [27].

Regarding safety and patient satisfaction, both home-based and clinic-based dPDT protocols showed excellent tolerability, with minimal pain and mild adverse reactions, which did not require any medical intervention (Table 2) [27]. No statistically significant differences between protocols were found in terms of pain, erythema, oedema, and crusting [27]. Three months after the treatment, participants in both groups were considerably satisfied with the overall outcomes (8.45 ± 0.93 vs. 8.67 ± 1.23, *p* > 0.05) [27].

## 5. Perspectives for Self-Applied Daylight Photodynamic Therapy Applications: Cutaneous Leishmaniasis and Hand Eczema

Studies have shown that ROS generated during PDT can directly interact with critical cellular components of *Leishmania* promastigotes, causing irreversible damage. For instance, proteins involved in cellular defence mechanisms, such as superoxide dismutase, exhibit altered expression levels post-PDT, reflecting the overwhelming oxidative stress imposed on the parasite. This oxidative insult disrupts membrane integrity, leading to mitochondrial dysfunction, lipid peroxidation, and loss of membrane potential, ultimately triggering apoptotic pathways [100,101,102].

Gene expression analysis has provided insights into the molecular events underlying PDT-induced cell death in *Leishmania*. For instance, photodynamic activation of curcumin significantly alters the expression of genes associated with ATP synthesis and oxidative stress responses, such as ATPase subunits and glucose-6-phosphate dehydrogenase. These changes impair the parasite’s energy metabolism and antioxidant defences, making it more susceptible to oxidative damage [101].

In addition to direct cytotoxic effects, PDT has been shown to modulate the host immune response, creating an environment less conducive to parasite survival. PDT can induce the upregulation of Th1-type cytokines, such as interferon-gamma IFN-γ, which enhances the host’s immune response against the parasite [103].

Other studies have demonstrated the potential of PDT in reducing parasite load and improving lesion healing in both murine models and human cases. For instance, dPDT has shown promise as a less invasive, self-administered treatment, being particularly effective against *Leishmania major* and *Leishmania tropica* [104]. In paediatric cases, dPDT has been well tolerated and has provided excellent cosmetic outcomes, emphasizing its potential as a patient-friendly alternative to conventional therapies [105].

In the context of SA-dPDT, a study involving 31 patients with CL was conducted. Fourteen patients were treated in the hospital with cPDT, while 17 treated themselves at home. Weekly sessions with MAL as the photosensitizer were performed, achieving a cure rate of 89% for SA-dPDT, compared to 89% for cPDT, showing no significant differences and demonstrating that fully home-based PDT protocols are a viable option for CL [104].

Despite these promising results, no PDT protocol is yet a gold standard treatment for CL. Its effectiveness is often limited by the depth of the lesion and the ability of the photosensitizer to penetrate infected tissues. Ongoing research is focused on optimizing PDT protocols, including the development of novel photosensitizers with enhanced tissue penetration and phototoxic properties [106].

Regarding the evidence on the use of SA-dPDT for inflammatory dermatoses, eight patients with chronic hand eczema were treated with SA-dPDT, showing moderate efficacy after four sessions spaced two weeks apart [107]. This study was published as a proof of concept, as PDT is not typically used for treating eczema.

## 6. Discussion and Conclusions

One of the primary advantages of cPDT and clinic-based dPDT is their straightforward application in a clinical setting under supervision. However, for elderly patients and their caregivers, attending a healthcare facility can lead to significant direct and indirect costs, limiting the accessibility of these treatments within community settings [97,108,109]. Although portable light source devices have been developed for home-based energy delivery with high clearance rates and good tolerability [110,111,112], their widespread use in the community remains impractical due to material and economic restraints.

Current consensus documents recommend that skin surface preparation, scaling removal, and photosensitizer application should be performed by a trained practitioners rather than by patients themselves [1,4,81,82,92,93,94,96]. The Australian position paper permits patients to remove the photosensitizer at home post-illumination [93]. However, evidence from real-world settings suggests that at-home skin preparation with keratolytic topical products can yield therapeutic outcomes comparable to standard dPDT protocols performed in a clinical setting [113].

Considering these factors, it stands clear that a certain subset of patients may benefit from SA-dPDT protocols, provided they first undergo dermatological evaluation and receive clear instructions on skin preparation, sunscreen use, and photosensitizer application and removal. This approach substantially increases the flexibility of dPDT, reducing patients’ time in the clinic for preparation and product removal. It also allows for a broader prescription of the treatment among individuals living in remote areas or with barriers to attending outpatient or inpatient settings, such as employment obligations, limited transportation options, or financial constraints [27,114]. Supporting this approach, a survey of patients previously treated with clinic-based dPDT revealed that 73% of participants would have rather been treated in a community-based setting [114].

Transitioning dPDT to a fully self-applied home-based topical therapy marks a fundamental shift from its traditional “in-patient” model [26]. Compared to other FC-targeted therapies, SA-dPDT stands out for its ease of application, minimal resulting pain, excellent tolerability, and shorter treatment duration (1 day vs. 5 days to 4 weeks) [26].

For practitioners prescribing dPDT, the most significant challenge is not in preparing the skin but in effectively communicating the need for patients to remain outdoors and receive adequate sunlight for the treatment to reach its full therapeutic potential. Because key parameters for illumination, such as temperature, time, and weather conditions, are complex for the general population to comprehend, instructions are often simplified in visual and easy-to-follow guides [1,4,81,82,92,93,94,96].

With recent advancements in patient education, no substantial barriers remain to the widespread adoption of home-based dPDT, as patients or caregivers can readily apply both keratolytic and photosensitizers to the affected areas.

In this regard, 98% of participants in the first large open-label trial closely followed the instructions, likely due to the simplicity and practicality of the protocol [28]. These findings were mirrored in a survey of 56 patients in the UK who had previously received clinic-based dPDT [114]. Following their clinical experience, 34% expressed a willingness to perform the treatment at home if provided with adequate support, and 15% felt confident in their ability to independently carry out the entire procedure [114].

In conclusion, SA-dPDT appears to be a promising therapeutic regimen for managing various skin conditions, particularly AK. Its ease of use, reported effectiveness, and tolerability makes SA-dPDT an attractive option for elderly and fragile patients, reducing the need for clinical visits. However, future research, including double-blind, large-scale RCTs with longer follow-up periods is essential to validate these preliminary findings.

## Figures and Tables

**Table 1:** Use of cPDT and dPDT in the treatment of AK and FC: recommendations of the European, German, American, and British guidelines [31,32,46,49].

		European Consensus-Based Interdisciplinary Guideline (EADO, EDF, EADV, UMS) [49]	German Guideline (Deutsche Dermatologische Gesellschaft) [32]	American Academy of Dermatology [31]	British Guideline [46]
**Year of publication**		2024	2023	2021	2017
**Conventional photodynamic therapy** (cPDT)					
**Redlight** (RL)					
**ALA-RL-PDT**	**Grade of recommendation**	A	B	Conditional	A
	**Quality of evidence**	1, 2	1	Low	1+
**MAL-RL-PDT**	**Grade of recommendation**	A	B	Not commercialized	A
	**Quality of evidence**	1, 2	1	Not commercialized	1+
**Bluelight** (BL)					
**ALA-BL-PDT**	**Grade of recommendation**	A	N/A	Conditional	A
	**Quality of evidence**	1, 2	N/A	Moderate	1+
**MAL-BL-PDT**	**Grade of recommendation**	A	N/A	Not commercialized	A
	**Quality of evidence**	1, 2	N/A	Not commercialized	1+
**Daylight photodynamic therapy** (dPDT)					
**ALA-dPDT**	**Grade of recommendation**	A	B	Conditional	A
	**Quality of evidence**	1, 2	1	Moderate	1+
**MAL-dPDT**	**Grade of recommendation**	A	B	Not commercialized	A
	**Quality of evidence**	1, 2	1	Not commercialized	1+

EADO: European Association of Dermato-Oncology; EDF: European Dermatology Forum; EADV: European Academy of Dermatology and Venereology; UMS: Union Européenne des Médecins Spécialistes.

**Table 2:** Self-applied daylight photodynamic therapy in the management of actinic keratoses: clinical studies [26,27,28,67,97].

	Levi et al. [67] *	Karrer et al. [28]	Garcia-Gil et al. [26]	O’Reilly et al. [97]	Saenz-Guirado et al. [27]
**Year of publication**	2018	2019	2022	2023	2024
**Type of study**	Retrospective cohort study	Open-label interventional multicentre study	Prospective observational open-label multicentre study	Prospective observational study	Single-blind randomised non-inferiority clinical trial
**n**	9	50	22	17	20 (11:9)
**Patients’ demographic variables**					
**Age** (years)	60.11 ± 14.93	73.4 ± 8	72.05 ± 6.96	Unspecified	76.45 ± 5.16 vs. 82.67 ± 4.41
**Male** (%)	66.67%	86%	90.5%	82%	100% vs. 88.9%
**Skin phototype**	Unspecified	II (70%)	II (50%), III (50%)	Unspecified	II (75%)
**Personal history of NMSC** (%)	Unspecified	Unspecified	45%	Unspecified	Unspecified
**Immunosuppressed** (%)	Unspecified	Unspecified	13.6%	Unspecified	0
**Control group**	N/A	N/A	N/A	N/A	Clinic-based dPDT
**Clinical indication**	Actinic cheilitis (AC)	Non-pigmented and non-hypertrophic actinic keratoses (AKs)	AK	AK	AK
**Anatomical location**	Lower lip (88.89%), upper lip (11.11%)	Face (60%), scalp (40%)	Face (50%), scalp (50%)	Head and neck	Scalp (68.63%), cheek (18.04%), forehead (13.33%)
**Baseline severity**	Unspecified	≥5 AK	≥5 AK	Unspecified	≥5 AK
*Overall number of lesions*	9	463	275	Unspecified	133
*Number of lesions per patient*	N/A	9.3 (6–29)	12.5 ± 4.34	Unspecified	12.09 ± 4.16 vs. 13.56 ± 3.79
*Olsen grade I*	N/A	Unassessed	47.3%	Unassessed	8.09 ± 3.7 vs. 10.56 ± 4.06
*Olsen grade II*	N/A	Unassessed	42.5%	Unassessed	4 ± 3.26 vs. 9.2 ± 3.97
*Olsen grade III*	N/A	Unassessed	10.2%	Unassessed	0
*AKASI*	N/A	Unassessed	4.99 ± 2.43	Unassessed	Unassessed
*AKQoL*	N/A	Unassessed	3.85 ± 4.63	Unassessed	Unassessed
**Previous treatments**	100%	69%	68.2%	Unspecified	Unspecified
*Cryotherapy*	66.67%	15%	36.4%		
*Surgical treatment*	11.11%		4.5%		
*Other ablative therapies*	11.11%				
*dPDT*			4.5%		
*Imiquimod*			22.7%		
*Diclofenac*		13%	27.3%		
*Ingenol mebutate*	0	19%			
*Other FC-targeting treatments*	11.11%				
**Exclusion criteria**	Unspecified	Unspecified	Unspecified	Unspecified	Diffuse involvement, immunosuppression, previous AK treatment 3 months before
**Protocol**					
**Photosensitizer**	MAL	MAL	MAL	MAL	BF-200 ALA
**Sunscreen prior to application of photosensitizer**	Organic filters (SPF20)	Organic filters (SPF50+), 15 min before MAL application	Organic filters	Organic filters	Organic filters
**Skin preparation**	First session: practitioner-performed gentle curettage/Subsequent sessions: skin preparation was left up to the patient	1–2 weeks before: practitioner-performed removal of the scales. Session: self-performed roughening with an abrasive pad	1 week before: urea 30% cream, once a day for 7 days	Unspecified	1 week before: 10% salicylic petrolatum, once a day for 7 days
**Country**	Israel	Germany	Spain	United Kingdom	Spain
**Onset of illumination**	Unspecified	30 min	30 min	30 min	30 min
**Sunlight exposure**	2.5 h (between 8 am and 11 pm)	2 h	2 h (between 11 a.m. and 5 p.m.)	2 h	2 h
**Practitioner-applied dPDT sessions**	1.44 (1–5)	0	0	0	1 (control group)
**SA-dPDT sessions**	1.44 (1–3)	1	1 (13.64%), 2 (86.36%)	4 (2–5)	1 (experimental group)
**Clinical outcomes**					
**Lesion clearance**	100%	3 months: 62%	9 months: 65.9%	3–6 months: 60% (≥75%), 7% (50–75%), 5 (<50%)	3 months: 82.1% vs. 78.35% (*p* > 0.05)
**Follow-up** (months)	31.44 ± 18.58	3	12	3–6 after last session	3
**AKASI**	Unassessed	Unassessed	12 months: 2.33 ± 1.01 (*p* = 0.0234)	Unspecified	Unassessed
**AKQoL**	Unassessed	Unassessed	12 months: 2.33 ± 2.69 (*p* > 0.05)	Unspecified	Unassessed
**Adverse effects**	100%	56%			Assessed 24 h after via telephone survey
**Pain**	N/A	Maximum pain (NRS: 0–10): 1 ± 1.4	NRS (0–10): 0.9 ± 2.32	Unassessed	VAS (1–10). During illumination: 2.27 ± 2.37 vs. 2.78 ± 2.17, *p* > 0.05; 24 h: 1.81 ± 1.9 vs. 1.22 ± 2.33, *p* > 0.05
**Erythema**	N/A	38%	72.7%	Unassessed	Patient-reported (24 h: 0–3): 1.09 ± 0.3 vs. 1.33 ± 1, *p* > 0.05; Physician reported (1 week: 0–3): 0.82 ± 0.6 vs. 0.67 ± 0.7, *p* > 0.05
**Skin burning sensation**	Unspecified	14%	Unspecified	Unassessed	Unassessed
**Oedema**	Unspecified	Unspecified	31.8%	Unassessed	Patient-reported (24 h: 0–3): 0.36 ± 0.5 vs. 0.22 ± 0.44, *p* > 0.05; Physician reported (1 week: 0–3): 0
**Crusting**	Unspecified	Unspecified	22.7%	Unassessed	Patient-reported (24 h: 0–3): 0.36 ± 0.5 vs. 0.22 ± 0.44, *p* > 0.05; Physician reported (1 week: 0–3): 0
**Vesicles**	Unspecified	Unspecified	5.3%	Unassessed	Unassessed
**Erosions**	Unspecified	Unspecified	5.3%	Unassessed	Unassessed
**Patients’ satisfaction**					
**Satisfaction with instructions**	Unassessed	98%	90.9%	Unassessed	Unassessed
**Satisfaction with overall outcome**	Unassessed	3 months: 96%	3 months: 72.7%	Unassessed	3 months (VAS): 8.45 ± 0.93 vs. 8.67 ± 1.23, *p* > 0.05

* The data of the column “Levi et al.” refer only to the patients who received at least one home-based dPDT session.

## Data Availability

No new data were created in this study. Data sharing is not applicable to this article.

## Abbreviations

AC	actinic cheilitis
AK	actinic keratosis
AKASI	Actinic Keratosis Area Severity Index
AK-FAS	Actinic Keratosis Field Assessment Scale
AKQoL	Actinic Keratosis Quality of Life
ALA	5-δ-aminolevulinic acid
BD	Bowen’s disease
BL	blue light
cPDT	conventional photodynamic therapy
CL	cutaneous leishmaniasis
DAMP	damage-associated molecular pattern
dPDT	daylight photodynamic therapy
ECM	extracellular matrix
FC	field cancerization
HP	haemtoporphyrin
HPD	haematoporphyrin derivative
MAL	methyl aminolevulinic acid
MMP	metalloproteinase
NMSC	nonmelanoma skin cancer
NRS	numeric rating scale
PF	protoporphyrin
PpIX	protoporphyrin IX
RL	red light
ROS	reactive oxygen species
S_0_	ground state
S_1_	singlet state
SA-dPDT	self-applied daylight photodynamic therapy
SCC	squamous cell carcinoma
RCT	randomised clinical trial
TERT	telomerase reverse transcriptase
Tn-C	tenascin-C
VAS	visual analogue scale
